# Occurrence of Oral Premalignant Lesions Among Tobacco Users in a Tribal Population: A Systematic Review and Meta-Analysis

**DOI:** 10.7759/cureus.47162

**Published:** 2023-10-16

**Authors:** Bhavana Gupta, Anish Gupta, Neha Singh, Rajeev Bhushan Singh, Vivek Gupta

**Affiliations:** 1 Department of Oral Pathology and Microbiology, Faculty of Dentistry, People's University, Bhopal, IND; 2 Department of Oral Pathology and Microbiology, People's Dental Academy, People's University, Bhopal, IND; 3 Periodontology, Private Practitioner, Ranchi, IND; 4 Department of Oral Pathology and Microbiology, Awadh Dental College and Hospital, Jamshedpur, IND; 5 Department of Periodontology and Oral Implantology, Dental Institute, Rajendra Institute of Medical Sciences (RIMS), Ranchi, IND

**Keywords:** oral submucous fibrosis, oral leukoplakia, potentially malignant lesions, oral mucosal lesions, smoking tobacco, chewing tobacco

## Abstract

This review aimed to comprehensively assess the association between tobacco use and oral health outcomes, specifically the presence of premalignant lesions (PMLs), through a synthesis of multiple assessments conducted in diverse populations. A systematic search of relevant literature was performed, and studies meeting the inclusion criteria were selected using appropriate Medical Subject Headings (MeSH) words and Boolean operators. Data from these studies was pooled and analysed using Review Manager 5.4 (The Cochrane Collaboration, The Nordic Cochrane Centre, Copenhagen). The Newcastle-Ottawa scale was used to assess the methodological quality of the studies included. The pooled analysis of the five selected papers revealed a significant correlation between tobacco use and an increased prevalence of PMLs among tobacco users. Tobacco users had an odds ratio of 15.22 (95% CI: 10.01-23.15) as compared to non-user cohorts, significant at p 0.0001. This comprehensive synthesis of assessments underscores the detrimental impact of tobacco use on oral health, particularly in terms of potentially malignant lesions. The findings emphasise the urgency of targeted public health interventions to address tobacco consumption and promote oral health awareness, especially in populations with high tobacco consumption rates. Standardisation of methodologies and representation of diverse populations in future research would strengthen the evidence base and facilitate more effective strategies to improve oral health outcomes globally.

## Introduction and background

Oral mucosal lesions (OMLs) represent a diverse group of pathological conditions affecting the oral cavity, ranging from benign to potentially malignant lesions [1]. Among the various risk factors contributing to the development of OMLs, tobacco use has emerged as a significant determinant, with both smoking and smokeless tobacco consumption linked to an increased prevalence of these lesions [2]. Tribal populations, characterised by distinct ethnic backgrounds and cultural practices, are particularly susceptible to the adverse effects of tobacco consumption, making them a crucial focus of investigation for public health initiatives [3]. Among smokeless tobacco users, the most commonly observed lesions include leukoplakia, erythroplakia, and oral submucous fibrosis (OSMF) [4]. Leukoplakia appears as white patches or plaques on the oral mucosa; erythroplakia manifests as red patches; and OSMF involves fibrotic changes in the oral submucosa leading to restricted mouth opening and difficulty chewing [4].

On the other hand, smoking tobacco is correlated with a greater risk of oral squamous cell carcinoma (OSCC) and various other OMLs, such as smoker's melanosis, nicotine stomatitis, and oral leukoplakia [5]. Smoker's melanosis presents as brown pigmentation in the oral mucosa, whereas nicotine stomatitis manifests as inflammation and white spots on the hard palate due to heat exposure from smoking [6]. The correlation between tobacco usage and OMLs varies based on the type and frequency of tobacco consumption as well as host susceptibility [7]. Smokeless tobacco users are particularly prone to developing leukoplakia and OSMF, whereas smoking tobacco increases the risk of OSCC and other smoking-associated lesions. The severity of OMLs can also be influenced by factors such as frequency, intensity, and duration of tobacco usage [8].

Numerous investigations have explored the tobacco consumption habits of tribal populations in India, revealing considerable prevalence rates of both smokeless and smoking tobacco use [9-12]. Studies have consistently demonstrated that a substantial proportion of tribal individuals engage in tobacco usage, with reports indicating that 56.2% of individuals aged over 15 years in tribal communities were found to be tobacco users in either smoking or smokeless form [9-11]. Moreover, a recent survey focusing on tribal communities revealed that 64.55% were tobacco users, among whom 29.1% used smoking tobacco, 63.4% used smokeless tobacco, and 7.5% engaged in dual consumption of both smoke and chewing form of tobacco [10]. Furthermore, findings from the same survey indicated that a significant percentage of tobacco users exhibited medium nicotine dependency, with rates of 82.75% and 53.57%, respectively [10]. These comprehensive reports underscore the prevalence and widespread nature of tobacco usage among tribal populations, drawing attention to the urgent need for targeted interventions and policy initiatives to address the challenges posed by tobacco-related health issues in these vulnerable communities [11-12]. Despite numerous studies exploring the association between tobacco use and premalignant lesions (PMLs), there remains a need for a comprehensive synthesis of evidence to understand the true prevalence of OMLs among tribal tobacco users. Hence, this systematic review was undertaken to answer the research question, "Is there a difference in the occurrence of pre-malignant lesions between tobacco users and non-tobacco users among the tribal population?"

## Review

Materials and methods

Eligibility Criteria

The review adhered to the rigorous guidelines of the Preferred Reporting Items for Systematic Reviews and Meta-Analyses (PRISMA) guidelines [13] and is registered with PROSPERO (CRD42023460957).

By clearly defining the PECOS protocol (population, exposure, comparator, outcome, study design), the review aimed to answer the research question, Does the habit of tobacco consumption increase the risk of oral mucosal lesions in tribal populations?" Population (P): The target population comprises individuals belonging to tribal origins of all age groups and geographic locations. Exposure (E): The exposure of interest is the consumption of tobacco. It includes both smoking (cigarettes, cigars, or pipes) and smokeless tobacco (chewing tobacco, snuff, or betel quid with tobacco) forms. Comparator (C): The comparator group included tribal individuals who did not consume tobacco in any form. Outcome (O): The primary outcome of interest was the prevalence or occurrence of oral mucosal lesions. The review focused on the prevalence of various types of mucosal lesions, such as leukoplakia, erythroplakia, and other PMLs, among tribal tobacco users. Study design (S): Clinical comparative studies (cross-sectional, cohort studies, and randomised control trials)were chosen.

Inclusion and Exclusion Criteria

Studies focusing on tribal populations that measured PMLs and tobacco use in these specific communities were included. Studies review's focussed only on tribal communities. Editorials, case reports, newsletters, and chronicles were not included. Articles not published in English were also excluded.

Search Strategy

The database search protocol identified relevant studies across eight different electronic databases. It was searched systematically using a combination of Medical Subject Headings (MeSH) keywords and Boolean operators, as represented by the search strings in Table 1. The databases included PubMed, Embase, Scopus, Web of Science, Cumulative Index to Nursing and Allied Health Literature (CINAHL), PsycINFO, Cochrane Library, and Google Scholar. The search strings for each database were constructed using the Boolean operators "AND" and "OR" to combine the MeSH keywords and their synonyms for the population, exposure, outcome, and study design. Truncation and wildcards were used where appropriate to ensure comprehensive retrieval of relevant studies.

**Table 1:** Search strings implemented across different databases CINAHL: Cumulative Index to Nursing and Allied Health Literature

Database	Search String
PubMed	("Tribal" OR "Indigenous" OR "Aboriginal" OR "Native") AND ("Population" OR "Community" OR "Ethnic Group") AND ("Tobacco Use" OR "Smoking" OR "Smokeless Tobacco" OR "Chewing Tobacco" OR "Tobacco Consumption") AND ("Oral Mucosal Lesions" OR "Oral Leukoplakia" OR "Oral Erythroplakia" OR "Potentially Malignant Lesions") AND ("Cross-Sectional Studies" OR "Cohort Studies" OR "Longitudinal Studies")
Embase	("Tribal" OR "Indigenous" OR "Aboriginal" OR "Native") AND ("Population" OR "Community" OR "Ethnic Group") AND ("Tobacco Use" OR "Smoking" OR "Smokeless Tobacco" OR "Chewing Tobacco" OR "Tobacco Consumption") AND ("Oral Mucosal Lesions" OR "Oral Leukoplakia" OR "Oral Erythroplakia" OR "Potentially Malignant Lesions") AND ("Cross-Sectional Studies" OR "Cohort Studies" OR "Longitudinal Studies")
Scopus	TITLE-ABS-KEY(("Tribal" OR "Indigenous" OR "Aboriginal" OR "Native") AND ("Population" OR "Community" OR "Ethnic Group")) AND TITLE-ABS-KEY(("Tobacco Use" OR "Smoking" OR "Smokeless Tobacco" OR "Chewing Tobacco" OR "Tobacco Consumption")) AND TITLE-ABS-KEY(("Oral Mucosal Lesions" OR "Oral Leukoplakia" OR "Oral Erythroplakia" OR "Potentially Malignant Lesions")) AND TITLE-ABS-KEY(("Cross-Sectional Studies" OR "Cohort Studies" OR "Longitudinal Studies"))
Web of Science	TS=("Tribal" OR "Indigenous" OR "Aboriginal" OR "Native") AND TS=("Population" OR "Community" OR "Ethnic Group") AND TS=("Tobacco Use" OR "Smoking" OR "Smokeless Tobacco" OR "Chewing Tobacco" OR "Tobacco Consumption") AND TS=("Oral Mucosal Lesions" OR "Oral Leukoplakia" OR "Oral Erythroplakia" OR "Potentially Malignant Lesions") AND TS=("Cross-Sectional Studies" OR "Cohort Studies" OR "Longitudinal Studies")
CINAHL	(Tribal OR Indigenous OR Aboriginal OR Native) AND (Population OR Community OR Ethnic Group) AND (Tobacco Use OR Smoking OR Smokeless Tobacco OR Chewing Tobacco OR Tobacco Consumption) AND (Oral Mucosal Lesions OR Oral Leukoplakia OR Oral Erythroplakia OR Potentially Malignant Lesions) AND (Cross-Sectional Studies OR Cohort Studies OR Longitudinal Studies)
PsycINFO	(Tribal OR Indigenous OR Aboriginal OR Native) AND (Population OR Community OR Ethnic Group) AND (Tobacco Use OR Smoking OR Smokeless Tobacco OR Chewing Tobacco OR Tobacco Consumption) AND (Oral Mucosal Lesions OR Oral Leukoplakia OR Oral Erythroplakia OR Potentially Malignant Lesions) AND (Cross-Sectional Studies OR Cohort Studies OR Longitudinal Studies)
Cochrane Library	("Tribal" OR "Indigenous" OR "Aboriginal" OR "Native") AND ("Population" OR "Community" OR "Ethnic Group") AND ("Tobacco Use" OR "Smoking" OR "Smokeless Tobacco" OR "Chewing Tobacco" OR "Tobacco Consumption") AND ("Oral Mucosal Lesions" OR "Oral Leukoplakia" OR "Oral Erythroplakia" OR "Potentially Malignant Lesions") AND ("Cross-Sectional Studies" OR "Cohort Studies" OR "Longitudinal Studies")
Google Scholar	("Tribal" OR "Indigenous" OR "Aboriginal" OR "Native") AND ("Population" OR "Community" OR "Ethnic Group") AND ("Tobacco Use" OR "Smoking" OR "Smokeless Tobacco" OR "Chewing Tobacco" OR "Tobacco Consumption") AND ("Oral Mucosal Lesions" OR "Oral Leukoplakia" OR "Oral Erythroplakia" OR "Potentially Malignant Lesions") AND ("Cross-Sectional Studies" OR "Cohort Studies" OR "Longitudinal Studies")

Data Extraction

The data extraction protocol for this investigation followed a systematic and standardised approach to retrieve relevant information from the included studies. Two independent reviewers meticulously extracted the data. The data extraction form encompassed elements such as study characteristics, participant demographics, tobacco use patterns, oral mucosal lesions identified, and prevalence data. To evaluate the reliability of data extraction, an interrater reliability test was conducted between the two reviewers. Using Cohen's kappa coefficient, the level of agreement between the reviewers was assessed. The results demonstrated excellent agreement, with a Cohen's kappa coefficient exceeding 0.75, affirming the consistency and accuracy of the data extraction process. Any discrepancies or inconsistencies in data extraction were carefully addressed during subsequent discussions between the reviewers. In instances where disagreements persisted, a third reviewer was consulted as an impartial adjudicator to ensure the accuracy and completeness of the extracted data.

Risk of Bias Assessment

The Newcastle-Ottawa Scale (NOS) [14] risk of bias assessment tool was used for the current review. The NOS encompasses a checklist that assesses the quality of a cross-sectional study in three domains: selection (this domain assesses how well the study population was selected); comparability (this domain assesses how well the exposed and unexposed groups were comparable at baseline); and outcome (this domain assesses how well the outcome was measured). The total score for the NOS is 9 points, with higher scores indicating a lower risk of bias. It was conducted by the same two reviewers.

Statistical Analysis

Review Manager 5.4 (The Cochrane Collaboration, The Nordic Cochrane Centre, Copenhagen) was used for synthesising quantitative analysis. A random effect model was applied considering the variability of the studies. The odds ratio at a 95% confidence interval was calculated. Forest plots were drawn to visualise the difference in PML occurrence between tobacco users and non-users.

Results

Study Characteristics

**Figure 1:**
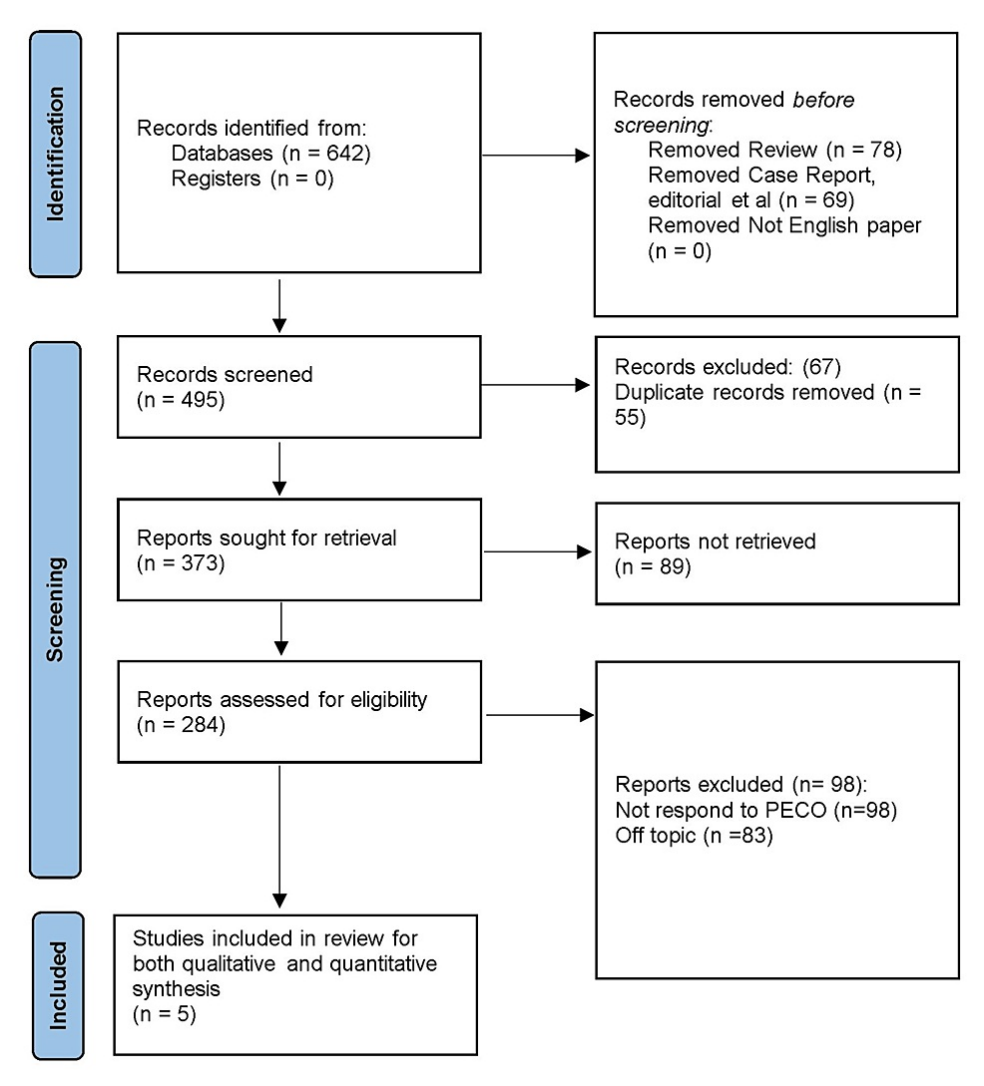
Graphical representation of the article inclusion process

In the beginning, a total of 642 records were identified from various databases. Among the identified records, 78 were removed as they were categorised as reviews, and 69 were excluded as they were either case reports, editorials, or other similar publications. After the initial screening, 495 records were deemed relevant and underwent further evaluation. Out of these, 55 duplicate records were removed, leaving 373 unique reports for retrieval. Unfortunately, 89 of these reports were not successfully retrieved, leaving 284 reports that were assessed for eligibility. During the assessment, 93 reports were excluded based on specific criteria. Among these, 98 reports did not respond to the PECOS framework, and 83 reports were considered off-topic for this review. Finally, the study selection process resulted in five studies [15-19] being included in the review as seen in Figure 1.

Main Findings

As evident from Table 2, all the included papers [15-19] with cross-sectional designs aim to explore the PML parameters within specific populations in India [15-18] and Taiwan [19]. The sample population ranged from 400 to 4542. The age range of the participants varied from >10 years to 84 years of age. No gender predilection was noted. Hence, studies showed a wide range of sample sizes, covering various age groups and gender ratios.

**Table 2:** Details on the population-related variables as seen in the chosen articles

Author	Year	Region	Protocol	Sample size (n)	Age range (in years)	Gender ratio
Agarwal and Bhattacharya [15]	2020	India	Cross-sectional	540	>10	258 males
Khanna et al. [16]	2012	India	Cross-sectional	411	11-85	233 males
Kumar et al. [17]	2015	India	Cross-sectional	4542	12-84	2272 males
Rajkuwar et al. [18]	2021	India	Cross-sectional	400	>12	152 males
Yang et al. [19]	2001	Taiwan	Cross-sectional	2059	>20	1048 males

Table 3 presents findings from all the selected papers [15-19] investigating the association between tobacco use and the presence of PMLs in different populations. Agarwal and Bhattacharya [15] used Pindborg’s criteria, and Rajkuwar et al. [18] used the WHO criteria for PML assessment. OSMF was quantified in all studies. Notably, synthesised results from all studies demonstrated a statistically significant association between consumers of tobacco and PMLs as compared to non-users.

**Table 3:** Specifics regarding tobacco habits and the presence of PMLs as shown in the chosen articles PML: Premalignant lesion

Author	Tobacco use status	Oral health assessment modality	PML type assessed	Types of oral lesions identified and their percentages
Agarwal and Bhattacharya [15]	54.8% of the sample size	Clinical examination and Pindborg’s classification	OSMF	47.4% of the total sample size experienced changes to their oral mucosa. Compared to 11.5% of non-users - 77% of the tobacco users had oral mucosal alterations. 59% of patients exhibited oral mucosa changes that were stage 1 in nature, manifesting as mucosal ulcers, erythema, melanotic pigmentation, and petechiae, while 35% were stage 2 in nature, with fibrous bands in 19.3% of cases. 5.8% of people had leukoplakia The age range with the most cases was 21–40 years, whereas oral lesions affected 53.6% of children between the ages of 10 and 19.
Khanna S [16]	53% of the sample size	Self-reported questionnaire and clinical examination	Leukoplakia, OSMF, BMS	Leukoplakia (10.7%), OSMF (6.3%), and Burning Mouth Syndrome (11.4%) were the most common OMLs observed. Males had a significantly greater percentage of OMLs as compared to females ( 23% versus 4%). 25% of the tribal members who got leukoplakia smoked, compared to 0.42% of the members who had leukoplakia but did not smoke. Also, compared to 3.06% of tribe members who had OSMF but no associated tobacco-related habit, 28.8% of those with OSMF reported using tobacco.
Kumar and Muniyandi [17]	65% of the sample size	Self-reported questionnaire and Visual evaluation	Oral leukoplakia, OSMF, oral ulcers	Oral leukoplakia was found to be present in 9.3% of the total sample size. OSMF was noted in 82 and oral ulcers in 43 Compared to non-users 2.5(%), tobacco users (11%) had considerably higher odds (OR = 4.8, p<0.001)of leukoplakia. Leukoplakia, however, was more prevalent (almost 22%) in tobacco chewers who also smoked.
Rajkuwar et al. [18]	88.25% of the sample size (smokeless tobacco)	Self-reported questionnaire and clinical examination (WHO oral health assessment form)	PMLs	Keratosis (60.2%) was the most common oral mucosal lesion followed by ulcers (10.7%) PMLs was noted in 27.84% of Smokeless tobacco users, 15.38% of smoked tobacco users and in 100% of combined users
Yang et al. [19]	39% of the sample size	Clinical examination	Oral leukoplakia and OSMF	Leukoplakia and OSMF were more common, with prevalence rates of 17.6% and 24%, respectively. Chewers + drinking alcohol has greater rates of OSF Chewers + alcohol consumers + Smoking had increased rates of leukoplakia Non chewers did not have any OSF

Risk of Bias Assessment

The NOS risk of bias assessment tool showed moderate quality in the studies included. All the studies fell between 5 and 9, thus ensuring good quality.

Metanalytic Results

**Figure 2:**
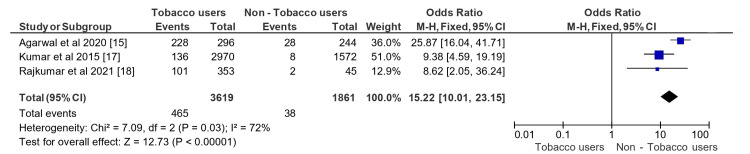
Forest plot depicting comparative evaluation of PMLs among tobacco users and non-users PMLs: Premalignant lesions

Three studies were included in the quantitative analysis, as seen in Figure 2.

**Figure 3:**
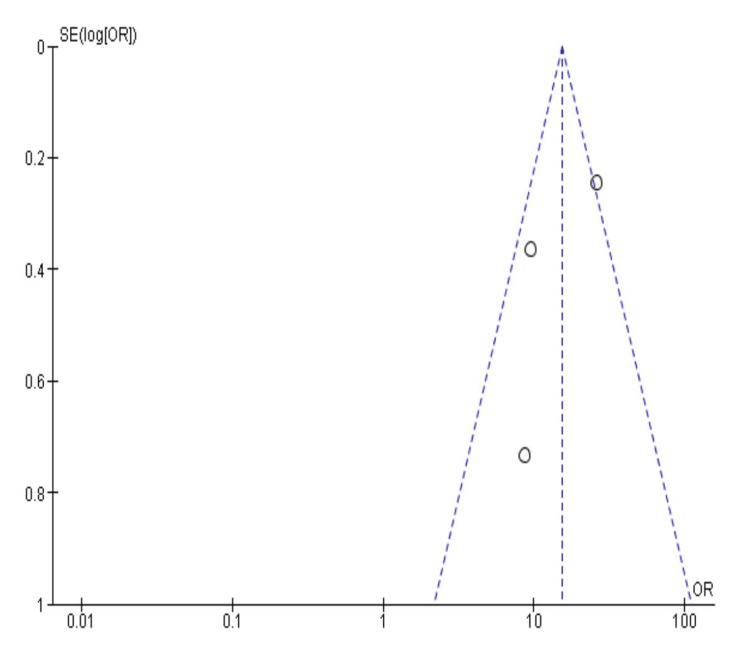
Funnel plot depicting publication bias for the analysis

The analysis included 3619 tobacco users, and 1861 non-users were analysed for the occurrence of PMLs. Tobacco users had an odds ratio of 15.22 (95% CI: 10.01-23.15) as compared to non-user cohorts, significant at p 0.0001. This signifies the greater risk of tobacco consumption in developing PMLs. The heterogeneity of the included studies accounted for 72%, prompting variability at the higher end. This variation could be attributed to the various methodological tools used, the variation in sample size, and the different PMLs measured. As seen in Figure 3, no publication bias was noted, suggesting a thorough search of the literature.

Discussion

Despite differences in sample sizes and assessment methods, all the included papers consistently demonstrate that tobacco use has a detrimental impact on PMLs. The prevalence of PMLs, including leukoplakia and keratosis, was notably higher among tobacco users. Both types of tobacco consumers were at greater risk of developing PMLs compared to non-users.

Interestingly, some studies specifically investigated the effects of chewing areca or betel quid, a habit prevalent in certain regions. Despite not containing tobacco, areca/betel quid chewing was strongly associated with a higher prevalence of PMLs, particularly leukoplakia, and OSMF. The assessments consistently emphasise the significance of addressing tobacco use as a critical risk factor for poor oral health and the development of PMLs. The synthesised findings highlight the necessity for comprehensive public health strategies and interventions aimed at reduction of tobacco consumption and promoting oral health awareness. The significance of these findings extends beyond individual studies, as the collective evidence underscores the urgent need for public health interventions aimed at curbing tobacco use and promoting oral health. The high prevalence of PMLs among tobacco users serves as a clear warning sign of potential oral cancer development, warranting targeted educational campaigns and cessation programmes to raise awareness and promote behaviour change.

Agarwal and Bhattacharya [15] collected data from two villages. A physician was consulted for a diagnosis. An interesting point in the study was that 36.5% of the population used Nas (dental snuff) as a dental cleaner. Adolescent girls used gudakhu, a mixture of jaggery and tobacco, to reduce menstrual pain. Hence, tobacco addiction started for reasons of social acceptability. Ignorance of the issue, along with the colourful marketing strategy of tobacco, resulted in sustained tobacco behaviour. Khanna S [16] reported the findings following field visits to villages where tribals habituated. The study revealed that tobacco habits were influenced by age, with tobacco consumption increasing with increasing age (significant at p 0.001). About 15.2% of people less than 20 years of age consumed tobacco as compared to 72% of people over 60 years of age. The methodological quality of the Khanna S study was compromised as no mention was made about the sample size determination, examiner, or indices used for clinical assessment. Also, tobacco history was not clearly elicited. Greater than half of the sample size were illiterates (n = 162). This could possibly justify the greater intake of tobacco among the sample. The tribals in the study of Kumar and Muniyandi [17] mostly belonged to lower socioeconomic strata. This, along with their refusal to undergo screening for oral diseases, could have accentuated the oral lesions. Rajkuwar et al. [18] used a translator for interviewing study participants. The study was done by a single examiner. Both genders were equally at risk of acquiring PMLs due to the significant use of smokeless tobacco (88.25%). An additional fact noted by the authors was the apprehension to participate in the study due to coronavirus disease 2019 (COVID-19). An individual in the study by Yang et al. [19] chewed 17.3 parts of areca or betel quid every day for an average of 24.4 years, making up 69.5% of the population. Areca or betel quid was used more by females (78.7%) than males (60.6%). There was a substantial correlation between chewing areca or betel quid and PMLs, despite the fact that Taiwanese areca or betel quid did not include any tobacco. Most of the tribals in their study were into mushroom farming.

Agarwal and Bhattacharya [15] reported that 54.8% of the sample size had a tobacco use status and underwent clinical examination. Among these participants, 47.4% experienced changes to their oral mucosa. Notably, the prevalence of oral mucosal alterations was significantly higher in tobacco users compared to non-users (77% vs. 11.5%). Among the tobacco users, 59% exhibited stage 1 oral mucosa changes, including mucosal ulcers and erythema, while 35% showed stage 2 changes, such as fibrous bands. Additionally, 5.8% of individuals had leukoplakia. The age range with the highest incidence of PMLs was 21-40 years, and children aged 10-19 years were also affected, with 53.6% having oral lesions. The study attributed the high frequency of tobacco use to the lack of preventive and promotional actions taken by health workers.

Several studies have been conducted in tribal communities to explore the association between tobacco habits and PMLs. These studies could not be included in the present review due to the absence of a comparator group. Deepa et al. [20] assessed tobacco use status through clinical examination and interviews in the Wayanad Tribes. The authors reported that 85.1% of the sample size had a tobacco use history. Among these participants, PMLs were prevalent, with 93 cases of leukoplakia, 38 cases of OSMF, two cases of OSCC, and 61 cases of Chewer's mucosa. All of these conditions were associated with tobacco use, indicating a significant relationship between tobacco use and the presence of PMLs in the assessed tribal group. Muthanandam et al. [21] performed clinical examinations on 65.23% of the sample size (nearly 80% of smokeless tobacco users) among tribes located in South India. A higher prevalence (48.3%) of precancerous lesions was noted. Among these, the labial mucosa had the second-highest number of lesions, with leathery alterations (28.3%) and white lesions (41.6%) being the most common. The study attributed the high rate of PMLs to tobacco use and behaviours connected to tobacco use in the population under assessment. Ramya et al. [22] conducted retrospective examinations on the Andaman and Nicobar tribes, revealing a prevalence rate of 28.8% for OSMF in the evaluated tribal group. Valsan et al. [23] used self-reported questionnaires and clinical examinations in Paniya tribes, indicating a prevalence of 3.6% for extraoral lesions and an overall prevalence of 4.52% for OMLs. The evaluated tribal population showed statistically low periodontal and oral health conditions.

In stark contrast to modern society, a substantial portion of the population falls under the lower socioeconomic strata, with specific social groups facing marginalisation [24]. Among these marginalised communities are tribal populations, which are generally known for their nomadic lifestyle and practices, including detrimental tobacco-related habits [25]. Despite their unique cultural and health characteristics, there is a paucity of scientific literature documenting the prevalence of oral lesions within this distinct tribal population [26].

Numerous studies have been conducted to explore the prevalence of oral precancerous lesions in different populations, shedding light on the distribution and gender-specific patterns of these lesions. One paper [9,26,27] reported a higher prevalence of OSMF and leukoplakia in males. Similarly, another article [28] observed a significantly higher prevalence of precancerous lesions among males (5.5%) compared to females in the general population of the Telangana region, India. Another paper [29] conducted a study, finding a prevalence of 27% for OMLs in tribal communities. A study [30] reported a 10.3% prevalence of leukoplakia among Baghi tribes. Once clinicians’ work [31] highlighted a much higher prevalence of precancerous oral lesions (42% among the underprivileged Paniya tribes), Additionally, they also recorded a prevalence of oral leukoplakia (12.9%) and OSMF (13.2%) among betel quid chewers in Paniya tribes [31]. On the contrary, Valsan et al. [23] reported a lower prevalence of OMLs (4.2%) among the tribal communities. An OML case detection rate of 2.5% was assessed in another cross-sectional survey [28]. These diverse findings highlight the importance of comprehending the epidemiology of oral precancerous lesions across various populations and emphasise the need for further research to comprehensively address this oral health concern [32-35].

Study limitations

Despite the valuable insights gained from the assessments presented in this review, there are several limitations that need to be considered when interpreting the findings. One notable limitation is the heterogeneity in the study designs and methodologies used across the selected studies. Variations in assessment protocols, sample sizes, and assessment periods may introduce inconsistencies and potential biases in the results. The lack of standardised approaches hinders direct comparisons, limiting the ability to draw definitive conclusions about the overall impact of tobacco use on oral health outcomes. Another limitation pertains to the potential for selection bias within the individual studies. Cross-sectional designs are susceptible to sampling biases, and the overrepresentation of males in the study populations might introduce gender-related biases in the observed associations between tobacco use and oral health outcomes. Additionally, the assessment of tobacco use status and oral health parameters relies on self-reporting and clinical examinations, both of which may be subject to recall bias and measurement errors. Furthermore, the lack of longitudinal data in the selected studies limits the ability to establish causality between tobacco use and oral health outcomes. While the associations between tobacco use and PMLs are consistently observed, longitudinal studies would provide more robust evidence on the temporal relationship and the potential cumulative effects of tobacco exposure on oral health.

## Conclusions

The synthesised qualitative and quantitative analysis reveals consistent associations between tobacco use and PMLs across tribal populations, emphasising the need for targeted public health interventions. The findings indicate that tobacco users have a significantly higher prevalence of oral leukoplakia and PMLs compared to non-users. The presence of leukoplakia was particularly pronounced among tobacco chewers and users of both smoked and smokeless forms of tobacco. Moreover, the combined use of tobacco and areca/betel quid chewing was linked to an elevated prevalence of PMLs. Furthermore, the assessments consistently identify poor oral health outcomes among tobacco users, with the observed indicators demonstrating the adverse effects of tobacco use on oral health. In our opinion, future research efforts should focus on standardising methodologies, expanding the representation of diverse populations, and conducting longitudinal studies to strengthen the evidence base and enhance the generalizability of the findings.
